# Drosophila Models Reveal Properties of Mutant Lamins That Give Rise to Distinct Diseases

**DOI:** 10.3390/cells12081142

**Published:** 2023-04-12

**Authors:** Sydney G. Walker, Christopher J. Langland, Jill Viles, Laura A. Hecker, Lori L. Wallrath

**Affiliations:** 1 Department of Biochemistry and Molecular Biology, Carver College of Medicine, University of Iowa, Iowa City, IA 52242, USA; 2 Independent Researcher, Gowrie, IA 50543, USA; 3 Department of Biology, Clarke University, Dubuque, IA 52001, USA

**Keywords:** cardiomyopathy, *Drosophila*, Emery–Dreifuss muscular dystrophy, familial partial lipodystrophy, Dunnigan type, intermediate filaments, laminopathy, lamins, nuclear envelope, nuclear pore

## Abstract

Mutations in the *LMNA* gene cause a collection of diseases known as laminopathies, including muscular dystrophies, lipodystrophies, and early-onset aging syndromes. The *LMNA* gene encodes A-type lamins, lamins A/C, intermediate filaments that form a meshwork underlying the inner nuclear membrane. Lamins have a conserved domain structure consisting of a head, coiled-coil rod, and C-terminal tail domain possessing an Ig-like fold. This study identified differences between two mutant lamins that cause distinct clinical diseases. One of the *LMNA* mutations encodes lamin A/C p.R527P and the other codes lamin A/C p.R482W, which are typically associated with muscular dystrophy and lipodystrophy, respectively. To determine how these mutations differentially affect muscle, we generated the equivalent mutations in the *Drosophila Lamin C (LamC)* gene, an orthologue of human *LMNA*. The muscle-specific expression of the R527P equivalent showed cytoplasmic aggregation of LamC, a reduced larval muscle size, decreased larval motility, and cardiac defects resulting in a reduced adult lifespan. By contrast, the muscle-specific expression of the R482W equivalent caused an abnormal nuclear shape without a change in larval muscle size, larval motility, and adult lifespan compared to controls. Collectively, these studies identified fundamental differences in the properties of mutant lamins that cause clinically distinct phenotypes, providing insights into disease mechanisms.

## 1. Introduction

Alterations in the expression of genes regulating muscle development can have either beneficial or detrimental effects on muscle function [1,2,3,4,5]. A classic example involves the *MSTN* gene, encoding myostatin, in which a dominant loss of function alleles causes muscle hypertrophy [6,7,8,9,10,11]. Breeding livestock with this mutation has been especially beneficial for the food industry, where producing pigs and cows with extremely large muscles is favorable [12,13,14,15]. Conversely, an overexpression of *MSTN* causes muscle wasting [16]. Thus, modulating the levels of a single protein can have opposing effects on muscle size. Such findings prompted us to investigate *LMNA* mutations discovered in two women with dramatically different muscle size that were featured in a ‘This American Life’ podcast titled “Something Only I Can See” [17]. The *LMNA* gene encodes A-type lamins, lamin A and C, intermediate filaments that form a meshwork on the inner side of the nuclear envelope [18,19,20]. Lamins have a conserved domain structure consisting of a globular head, coiled-coil rod, and tail domain [21].

The podcast tells the story of Jill Viles, who is heterozygous for a point mutation in the *LMNA* gene that gives rise to p.R527P, altering the Ig-like fold domain. Prior to the discovery of the mutation in *LMNA*, physicians failed to classify her muscle disease. Through her own investigations, she diagnosed herself with autosomal dominant Emery-Dreifuss muscular dystrophy type 2 (EDMD2), which was later confirmed by DNA sequence analysis. EDMD2 is characterized by muscle weakness and wasting, especially of the proximal arm, shoulders, and lower leg. In addition, muscle loss is often accompanied by a triad of contractures that include the Achilles tendon, elbows, and neck. EMDM2 is often accompanied by cardiomyopathy with conduction defects [22,23,24]. Exacerbating the thinness of Jill’s musculature, she has symptoms that overlap with familial partial lipodystrophy type 2 (FPLD2), characterized by metabolic alterations that result in the loss of subcutaneous adipose tissue and sometimes insulin-resistant diabetes [25,26,27]. The podcast also describes Priscilla Lopes-Schliep, who is heterozygous for a point mutation in *LMNA* that causes p.R482W, also altering the Ig-like fold domain. She is a 2008 Canadian Olympic bronze medalist in the hurdles who was repeatedly accused of taking performance-enhancing drugs due to the large size and prominent definition of her muscles. These accusations were later attributed to her diagnosis of FPLD2, with prominent musculature likely due to a loss of subcutaneous fat. While the muscular physique of these two women is in stark contrast to one another, they have common physical features, such as prominent veins in their arms and legs due to a loss of adipose tissue. These divergent skeletal muscle phenotypes call to question how these two mutations in *LMNA* have opposing effects on muscle size.

Over 400 mutations in *LMNA* have been linked to diseases collectively called laminopathies, the majority affecting muscle [28,29,30]. Most *LMNA* DNA lesions are point mutations that give rise to single amino acid substitutions throughout the head, rod, and tail domain, which possess an Ig-like fold [31,32]. Several studies have attempted to establish a correlation between the genotype and disease phenotype; however, such correlations are limited [6,31,32,33,34,35,36,37]. In addition, the same amino acid substitution can give rise to clinically distinct diseases, even among closely related individuals [31,34,38]. In fact, this is the case for the mutations identified in Jill and Priscilla in which individuals can have either muscular dystrophy, lipodystrophy, or both [39,40,41]. Thus, the genetic background of an individual is considered to play a role in modifying laminopathy phenotypes. These confounding effects of genetic background differences make it challenging to identify defects associated with specific *LMNA* mutations.

The cases of Jill and Priscilla raise many questions about laminopathies in general. Could an amino acid substitution at one site in the lamin A/C protein cause skeletal muscle hypertrophy, while at another site cause muscle atrophy? Are the clinical distinctions between these two women due to the specific *LMNA* mutations or their genetic background? In other words, does Priscilla’s genetic background possess suppressor(s) of skeletal muscle disease, or does Jill’s genetic background possess enhancer(s) of the skeletal muscle disease? To address these questions, we aimed to functionally test these two *LMNA* mutations in a defined genetic background. We modeled each mutation into the *Drosophila melanogaster Lamin C* (*LamC*) gene, an orthologue of *LMNA*, and expressed the mutant *LamC* in muscle tissue. *Drosophila* serves as a valuable organism for modeling many types of human diseases, including muscular dystrophies [42,43,44,45,46,47,48,49,50,51,52]. The use of *Drosophila* allows for comparisons of the effects of specific mutations in a nearly identical genetic background. We show that the *Drosophila* models recapitulate the muscle phenotypes exhibited by Jill and Priscilla. These findings support the hypothesis that the divergent muscle phenotypes are due to the specific amino acid substitutions rather than genetic background differences. When expressed in larval body wall muscles, the mutant lamins exhibit strikingly different patterns of localization and have opposing effects on the nuclear envelope shape and larval motility. These changes have effects on the intracellular distribution of additional nuclear envelope proteins and genomic DNA organization. The expression of these mutant lamins in cardiac tissue results in dramatically different adult median survival lifespans. Collectively, our findings revealed differences in the properties of two lamin Ig-like fold domain mutants that provided insights into the molecular mechanisms associated with skeletal muscle laminopathies.

## 2. Materials and Methods

### 2.1. Drosophila Stocks

*Drosophila* stocks were maintained at 25 °C on a sucrose and cornmeal-based medium [53]. A full-length *LamC* cDNA (Gold Clone, *Drosophila* Genomics Resource Center) was used for site-directed mutagenesis (QuickChange, Agilent, Santa Clara, CA, USA) to generate base changes predicted to encode LamC p.K521W and p.R564P. The resulting mutated versions of *LamC* were cloned downstream of an upstream activating sequence (UAS) and a minimal promoter in the pUAST *Drosophila* transformation vector (*Drosophila* Genomic Resource Center, Bloomington, IN, USA). Transgenic stocks were generated by standard P-element transformation procedures (BestGene Inc., Chino Hills, CA, USA) [54]. Homozygous stocks were generated via crosses of flies heterozygous for the transgene to stock-bearing balancer (multiply inverted) chromosomes possessing the second and third chromosome markers *Curly* and *Stubble*, respectively (stock #2475, Bloomington Stock Center, Bloomington, IN, USA). The resulting progeny were interbred to recover homozygous flies lacking the marked balancer chromosomes.

### 2.2. Western Analysis

Protein extracts were generated from two hand-dissected third instar larval muscle filets. The samples were lysed using 2X Laemmli Buffer (125 mM Tris HCL, 20% glycerol, 4% SDS, 0.005% bromophenol blue, final pH 6.8) and 20 mM DTT. Samples were boiled for five minutes and then centrifuged at 15,000 rpm for five minutes at room temperature. The supernatants were collected, 30 µL of each sample was loaded onto a NuPage 4-12% BisTris gradient gel in 1X MES/SDS, and the proteins were separated by size via electrophoresis. Proteins were transferred to a nitrocellulose membrane and blocked in 5% BSA. The membranes were incubated with an antibody to GAPDH (1:10,000 dilution, Developmental Studies Hybridoma Bank, University of Iowa, Iowa City, IA, USA) and LamC (1:2000 dilution, Developmental Studies Hybridoma Bank, University of Iowa, Iowa City, IA) diluted in 1% BSA/TBS-T. The membranes were incubated overnight at 4 °C on a rotating platform, washed three times with TBS-T, and incubated with anti-mouse (Dylight 800 LI-COR, 1:10,000) and anti-rabbit (Dylight 680 LI-COR, 1:10,000 dilution) secondary antibodies diluted in 1% BSA/TBS-T at room temperature for an hour. The membranes were washed three times in TBS-T and twice with TBS and then imaged using LI-COR Odyssey CLx and quantified using ImageStudioLite (v5.2.5, LI-COR software, Lincoln, NE, USA). LamC protein levels were normalized to GAPDH levels and made relative to the level of LamC in the wild-type sample. The results from three independent biological samples were averaged and plotted. Statistical significance was determined using a one-way ANOVA multiple comparisons analysis followed by Dunnett’s correction (GraphPad Prism version 9.5.0, GraphPad Software, San Diego, CA, USA).

### 2.3. Immunohistochemistry of Drosophila Larval Tissues

For larval body wall muscle and fat body tissue immunostainings, third instar larvae with tissue-specific expression of either wild-type or mutant LamC were dissected and fixed in 4% formaldehyde solution. The larval muscle filets and fat body tissues were washed with muscle buffer (128 mM NaCl, 5 mM Hepes pH 7.4, 2 mM KCl, 35 mM Sucrose) twice and placed in Eppendorf tubes containing 1X PBS. The larval body filets were then washed three times with 1X PBS and three times with permeabilization buffer (1X PBS, 0.5% TX100, 5 mM MgCl_2_). The tissues were stained using Texas-Red Phalloidin (1:400 dilution, Invitrogen, Waltham, MA, USA) and incubated with mouse primary antibodies against LamC (1:200 dilution, Developmental Studies Hybridoma Bank, University of Iowa, Iowa City, IA, USA), lamin Dm_0_ (1:400 dilution, Developmental Studies Hybridoma Bank, University of Iowa, Iowa City, IA, USA), goat antibodies Otefin (1:500) against [55], rabbit antibodies against TMEM43 (1:800 dilution, Thermo Fisher Scientific, Waltham, MA, USA), and mouse antibodies against FG-repeat containing nuclear pore proteins (1:2,000 dilution, Covance, Princeton, NJ, USA). All antibodies were diluted in permeabilization buffer containing 0.5% boiled/filtered fish skin gelatin. The tissues with the primary antibodies were incubated overnight at 4 °C. Following incubation, tissues were washed three times with permeabilization buffer and incubated at room temperature for two hours with either an anti-mouse or anti-goat secondary antibody Alexa-Fluor 488 (1:400 dilution, Invitrogen, Waltham, MA, USA) diluted in permeabilization buffer containing 0.5% boiled/filtered fish skin gelatin. After incubation, the tissues were washed three times with permeabilization buffer and three times with 1X PBS. The larval body filets were mounted on slides using Vectashield Antifade Mounting Medium with DAPI (Vector Laboratories, Burlingame, CA, USA) and examined using either a Leica DMLB fluorescent microscope with 40X oil objective or a Leica Thunder microscope with a 63X oil objective.

Two-channel microscopic fluorescent images of larval body wall muscles were analyzed to determine the relationship between nuclear envelope protein and DNA localization. The images were split into separate channels, one containing the signal for DAPI (blue) and the other containing the signal for either LamC or lamDm_0_ (green). The background was subtracted from each channel using the background subtraction tool (Fiji; [56]). In the green channel, regions of interest (ROIs) were defined by tracing nuclei with the polygon selection tool in Fiji [56] and adding the selected image to the ROI manager. Using the multi-crop function in the ROI manager, the ROIs were cropped from green and blue channels. The cropped images were analyzed for co-localization of signal in the green and blue channels using the Just Another Co-localization Plugin (JACoP) [57]. The Manders’ coefficient was calculated using JACoP for the fraction of staining in the green channel that overlaps with staining in the blue channel. Seven to thirty-eight nuclei from three individual larvae per genotype were analyzed. Statistical significance was determined using a one-way ANOVA multiple comparisons analysis followed by Dunnett’s correction (GraphPad Prism version 9.5.0, GraphPad Software, San Diego, CA, USA).

For adult heart immunostaining, seven-day-old adults with heart-specific expression of either wild-type or mutant LamC were dissected in ADH buffer (108 mM Na^+^, 5 mM K+, 2 mM Ca^2+^, 8 mM MgCl_2_, 1 mM NaH_2_PO_4_, 4 mM NaHCO_3_, 10 mM sucrose, 5 mM trehalose, 5 mM HEPES) and stained according to published procedures [58]. The hearts were placed in a shallow well plate and briefly washed with relaxing buffer (ADH that contains 10 mM EGTA) and then fixed in 4% formaldehyde solution for 20 min. The hearts were washed three times for 10 min with PBSTX (PBS containing 0.1% Triton-X-100) and were stained with anti-LamC antibodies (1:200 dilution, Developmental Studies Hybridoma Bank, University of Iowa, Iowa City, IA, USA) and Texas-Red Phalloidin (1:400 dilution, Invitrogen, Waltham, MA). All antibodies were diluted in PBSTX, and the hearts were incubated overnight at 4 °C. The adult hearts were washed three times for ten minutes in PBSTX followed by incubation with antibody Alexa-Fluor 488 (1:400 dilution, Invitrogen, Waltham, MA, USA) for 60 min at room temperature. The adult hearts were washed three times for 10 min in PBSTX with a final wash of PBS for ten minutes. Two coverslips were adhered 15 mm apart on a slide with a drop of Vectashield Mounting Medium with DAPI (Vector Laboratories, Burlingame, CA, USA). The hearts were placed facing downward on a drop of Vectashield Mounting Medium with DAPI on a third cover slip. The coverslip containing the hearts was flipped upside down and placed as a bridge between the 15 mm gap. The slides were examined using a Leica Thunder microscope with a 63X oil objective.

### 2.4. Oil Red O Staining of Adipose Tissue

Fat bodies were dissected from third instar larvae and stained with Oil Red O (Sigma-Aldrich St. Louis, MO, USA) as previously described [59]. Briefly, the fat bodies were dissected in 1X PBS, fixed in 4% paraformaldehyde for 20 min at room temperature, and washed twice with 1X PBS. The fat bodies were stained with a working solution of six parts 0.1% Oil Red O in isopropanol and four parts distilled water for 30 min at room temperature. Tissues were washed once in distilled water with 60% isopropanol, and then 1X PBS. The fat bodies were mounted on slides using Vectashield Antifade Mounting Medium with DAPI (Vector Laboratories, Burlingame, CA, USA) and examined using a Leica Thunder microscope with a 63X oil objective. Quantification of the lipid droplets was performed using Fiji [56]. Single-channel images were cropped to an area of 4936 µm^2^ and a color threshold was set to highlight the lipid droplets. Lipid droplets were measured using the analyze particles function. The percentage area of lipid droplets was determined. The mean and standard deviation were calculated and plotted using Graphpad Prism (v9.2.0, Graphpad Software, La Jolla, CA, USA). Statistical significance was determined using a one-way ANOVA multiple comparisons analysis followed by Dunnett’s correction (GraphPad Prism version 9.5.0, GraphPad Software, San Diego, CA, USA).

### 2.5. Larval Body Wall Muscle and Adipose Cell Size Measurements

Third instar larvae were hand-dissected by making a longitudinal incision and removing the internal organs. The resulting larval body wall muscle filet was fixed in 4% formaldehyde solution. The muscle filets were washed with muscle buffer twice and placed in microfuge tubes containing 1X PBS. The larval body wall muscle filets were washed three times with 1X PBS and three times with permeabilization buffer (1X PBS, 0.5% TX100, 5 mM MgCl_2_). Filets were stained using Texas-Red phalloidin (1:400 dilution, Invitrogen, Waltham, MA, USA). The larval body wall muscle filets were mounted on slides using Vectashield Antifade Mounting Medium with DAPI (Vector Laboratories, Newark, CA, USA) and examined using Leica Thunder microscope tiling function with a 20X dry objective. Measurements of larval body wall muscles were quantified using Fiji [56]. Straight lines were drawn to measure the length and width of each respective muscle and measured in micrometers using the measure tool. Statistical significance was determined using a one-way ANOVA multiple comparisons followed by Dunnett’s correction (GraphPad Prism version 9.5.0, GraphPad Software, San Diego, CA, USA).

Fat bodies were dissected from third instar larvae, fixed, and stained with phallodin, DAPI, and anti-LamC antibodies. Phallodin marks the periphery of the fat body cells. To measure the area of fat body cells, the freehand tool available in Fiji [56] was used to outline the cells. The area of each cell (μm) was measured using the measure tool available in Fiji [56]. Ten to thirty-two fat body cells from four independent larvae were analyzed per genotype. The mean, standard deviation, and statistical significance were determined using Graphpad Prism (v9.2.0, Graphpad Software, La Jolla, CA, USA). Statistical significance was determined using a one-way ANOVA multiple comparisons with Tukey’s correction (GraphPad Prism version 9.5.0, GraphPad Software, San Diego, CA, USA).

### 2.6. Larval Motility Assays

Third instar larvae with muscle-specific expression of either wild-type or mutant LamC were placed on a 15 cm petri dish containing a thin layer of room-temperature water. After five minutes of equilibration, five larvae per genotype were transferred to a second 15 cm petri dish containing a thin layer of water. A piece of graph paper with millimeter divisions was placed under the petri dish. A two-minute video recording of the larvae was captured using a cell phone [60]. Larval velocity was calculated by examining the larval distance crawled over a 10 s time interval and body contractions were counted during the same 10 s time interval. The distance crawled per contraction was calculated by dividing the distance traveled during the 10 s interval by number of larval contractions. Larvae (nine to nineteen) were assayed per genotype. Statistical significance was determined using a one-way ANOVA multiple-comparisons analysis followed by Dunnett’s correction (GraphPad Prism version 9.5.0, GraphPad Software, San Diego, CA, USA).

### 2.7. Adult Viability Measurements

Flies with wild-type and mutant *LamC* transgenes were crossed to flies possessing the muscle-specific Gal4 driver called *C57* [61]. After five days, the adults were removed from the vials and progeny were allowed to develop. The resulting dead pupae and live adults were counted. The percentage adult viability was calculated as $\frac{\#\;living\;adults}{\#\;living\;adults+\#\;dead\;pupae}\times$ 100. The total number of progeny ranged from 255–326 per genotype. Statistical significance was determined using the Fisher’s exact test (GraphPad Prism version 9.5.0, GraphPad Software, San Diego, CA, USA).

Adult flies possessing either wild-type or mutant *LamC* transgenes were crossed to adults possessing a cardiac-specific *dHand4.2* Gal4 driver [62] (gift of G. Melkani, U. Alabama at Birmingham, AL, USA). After five days, the adults were removed from the vials. Newly emerged adults were counted daily and placed in fresh vials every third day. The median survival time and the statistical analysis using the Mantel–Cox test were calculated using GraphPad Prism (version 9.5.0, GraphPad Software, San Diego, CA, USA).

### 2.8. Statistical Analysis

Values for larval velocity, distance per larval contraction, muscle length and width, and western analyses were entered into Graphpad Prism (version 9.5.0, GraphPad Software, San Diego, CA, USA). The software was used to plot the mean and standard deviation, as well as statistical significance using a one-way ANOVA multiple-comparisons test followed by Dunnett’s or a Tukey’s correction (version 9.5.0, GraphPad Software, San Diego, CA, USA). Significance of the differences in percentage adult viability between each genotype and the control was determined using the Fisher’s exact test (version 9.5.0, GraphPad Software, San Diego, CA, USA). The extent of colocalization of nuclear envelope protein antibody staining with DAPI was determined using the Manders’ coefficient, converted to a percentage, and statistical differences were determined by a one-way ANOVA analysis followed by Dunnett’s multiple comparisons test (version 9.5.0, GraphPad Software, San Diego, CA, USA). In addition, the GraphPad Software was used to plot survival data, determine the median survival time, and perform the Mantel–Cox statistical significance test. Differences between conditions were considered significant at levels of * *p* < 0.05, ** *p* < 0.01, *** *p* < 0.001, and **** *p* < 0.0001. Non-statistical differences between conditions were marked as not significant (ns).

## 3. Results

### 3.1. Mutant Lamins Have Different Patterns of Localization and Effects on Myonuclear Morphology

**Figure 1:**
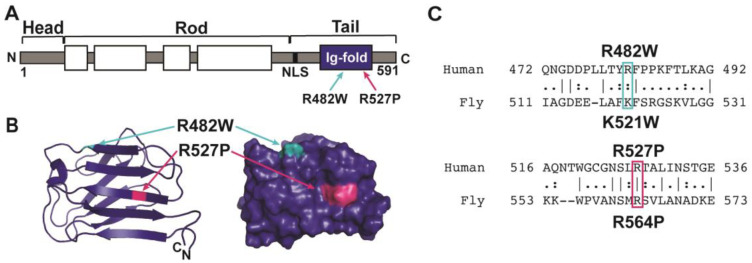
A-type lamins have three conserved protein domains. (**A**) Diagram of lamin A/C showing the head, coiled-coil rod, and tail domain that possesses a nuclear localization sequence (NLS) and an Ig-like fold domain. (**B**) Ribbon diagram of the Ig-like fold domain (PDB: 1IVT) showing the position of the two amino acid substitutions studied here (arrows). (**C**) Amino acid sequence comparison of human lamin A/C and *Drosophila* LamC is shown for the sequences surrounding the amino acid substitutions studied here. Amino acid R482 in human lamin A/C is a conserved K521 in *Drosophila* LamC. Amino acid R527 in human lamin A/C is identical between the two species and corresponds to R564 in *Drosophila* LamC.

Lamin A/C R482 and R527 map to the Ig-like fold domain (PBD: 1IVT) (Figure 1A,B) [63]. Specifically, R482 resides in the middle of β-sheet 5C’; R527 resides at the C-terminal end of β-sheet 8F [63]. To understand the effects of the R482W and the R527P amino acid substitutions on muscle function, we took an in vivo approach by generating transgenic *Drosophila.* The *Drosophila* models allowed us to test the effect of each amino acid substitution on muscle physiology in a nearly identical genetic background. Thus, phenotypic differences can be attributed to a specific amino acid substitution and not to background modifier genes.

The *Drosophila Lamin C* (*LamC*) gene is an orthologue of human *LMNA* and has been used to model laminopathies [31,49,50,64]. The *Drosophila* LamC protein and human lamin A/C have a highly similar domain structure with a 35% amino acid sequence identity and 54% similarity throughout. Amino acid R527 in human lamin A/C corresponds to amino acid R564 in *Drosophila* LamC, and the amino acid residue R482 in human lamin A/C corresponds to the highly conserved amino acid K521 in *Drosophila* LamC (Figure 1C). We generated transgenic flies expressing wild-type and mutant LamC under the control of an upstream activating sequence (UAS), which allows for tissue-specific inducible expression via the Gal4 transcription factor [65].

Adult flies possessing either a wild-type or mutant *LamC* transgene were mated to adults possessing the *C57* Gal4 driver that expresses the Gal4 transcription factor specifically in larval body wall muscles [65,66,67]. The *LamC* transgenes were not fused to sequences encoding a protein tag since tags often cause lamins to be mislocalized and/or aggregate [68,69,70]. Therefore, the antibodies against LamC do not discriminate between LamC produced from the transgenes and that from the endogenous *LamC* gene. A quantitative Western blot analysis was performed on larval body wall muscle protein extracts from the transgenic lines (Figure S1A,B). These data showed slightly elevated levels of total LamC levels in larvae expressing the wild-type *LamC t*ransgene compared to larvae lacking a transgene (Figure S1C). LamC levels in larvae expressing LamC K521W (transgenic line 2-M4) and LamC R564P (transgenic line 67F) showed no statistical difference in comparison to each other and to levels in larvae expressing wild-type LamC (Figure S1C). By contrast, other transgenic lines, such as LamC K521W (2-M5), showed approximately two to three-fold increased levels of expression. Therefore, transgenic lines LamC K521W (2-M4) and R564P (67F) were selected for all subsequent experiments.

**Figure 2:**
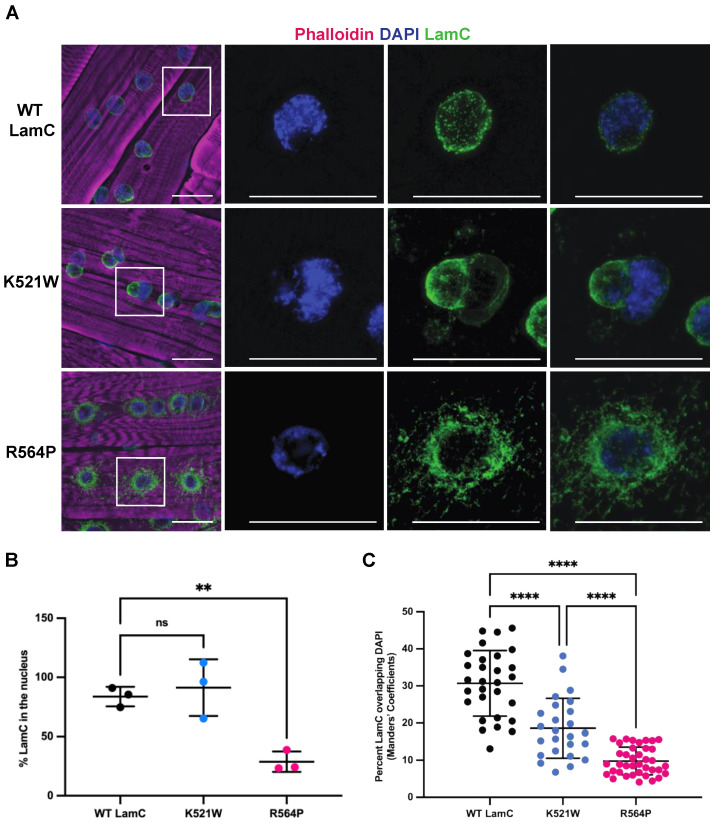
LamC K521W and R564P show different patterns of LamC immunostaining. (**A**) Larval body wall muscles expressing either wild-type or mutant LamC were stained with phalloidin (magenta), DAPI (blue), and antibodies to LamC (green). Note the abnormal nuclear morphology caused by LamC K521W and the cytoplasmic lamin aggregation caused by LamC R564P. The white box outlines the magnified region shown on the right. The scale bar represents 30 µm. (**B**) A graph of the average percent of nuclear LamC in each genotype is shown. The subcellular location of LamC was quantified based on the intensity of immunofluorescent staining in the nucleus compared to the total intensity from three-channel microscopy images of larval body wall muscles. (**C**) A graph of the quantification of the overlap of LamC and DAPI in larval body wall muscles is shown. The Manders’ coefficient was calculated using JACoP for the fraction of staining in the green channel (LamC) that overlaps with staining in the blue channel (DAPI). A total of 25 to 38 nuclei from three individual larvae per genotype were analyzed. Error bars represent the standard deviation of the mean. The values in the graph are expressed as mean ± standard deviation. A total of 15-34 nuclei were analyzed from three larvae per genotype. For panels (**B**,**C**), statistical significance was determined using a one-way ANOVA multiple comparisons analysis followed by Dunnett’s correction (GraphPad Prism version 9.5.0, GraphPad Software, San Diego, CA, USA) and is indicated by: not significant (ns), *p* > 0.05; **, *p* < 0.01; ****, *p* < 0.0001.

To determine the localization pattern of LamC, larvae body wall muscles were used for immunohistochemistry. Larval body wall muscles have similar developmental and physiological features to human skeletal muscles [71]. The multinucleated muscle fibers are arranged in a stereotypical pattern and connect to epidermal cells by tendons [72,73,74]. The contraction of the larval body wall muscles is responsible for larval motility and morphogenesis during the pupal stage [75,76]. The immunostaining of larval muscles expressing each mutant lamin showed strikingly different patterns of localization (Figure 2A). These abnormal patterns of LamC localization were dependent upon the expression of GAL4 and were therefore not a result of the transgene insertion (Figure S2). Muscles expressing wild-type LamC had spherical nuclei like that of the non-transgenic control. By contrast, expression of LamC K521W produced lobulated nuclei. This atypical nuclear shape suggested that the underlying nuclear lamina was not properly assembled. LamC R564P showed spherical nuclei; however, there was extensive LamC staining in the cytoplasm: only an average of 29% of the LamC was nuclear (Figure 2A,B). The lack of nuclear lobulations in muscles expressing LamC R564P might be because little if any mutant LamC enters the nucleus (Figure 2A). By contrast, muscles expressing wild-type LamC and LamC K521W showed 84% and 91% of LamC within the nucleus, respectively (Figure 2A,B). Thus, the two mutant lamins conferred different patterns of localization and distinct nuclear morphologies.

Lamins play a role in the three-dimensional organization of genomic DNA within the nucleus [77,78,79,80]. Lamins interact with DNA to form lamin-associated domains (LADs) that play a role in gene regulation [81,82,83]. Given the dramatic changes in LamC localization, we investigated the overlap between the LamC antibody and DAPI staining. In muscles expressing wild-type LamC, an average of 31% of the LamC staining co-localized with DAPI (Figure 2C). By contrast, muscles expressing LamC K521W and R564P showed an average of 19% and 10% colocalization with DAPI (Figure 2C). A reduction in the overlap between LamC and DAPI in muscles expressing LamC K521W is due to the nuclear lobulations in which some lobules are enriched for LamC and have a paucity of DAPI staining, whereas others are enriched for DAPI and have limited LamC antibody staining (Figure 2A,C). A reduction in the overlap between LamC staining and DAPI in muscles expressing LamC R564P is due to the extensive non-nuclear localization of LamC (Figure 2A,C). This implies that the altered lamina meshwork formed by LamC K521W decreases the association of the genomic DNA with the nuclear periphery, a compartment known to induce gene silencing [84,85].

### 3.2. Mutant Lamins Have Different Effects on Nuclear Envelope Protein Localization

B-type lamins are expressed in nearly all cell types, whereas A-type lamins are only expressed upon differentiation [86]. A- and B-type lamins form distinct meshworks underlying the inner nuclear membrane [87,88]. B-type lamin forms an outer concentric ring located immediately adjacent to the inner nuclear membrane. A concentric ring of A-type is slightly more interior and spatially distinct from the B-type concentric ring [87]. It is not well understood how defects in one type of lamin might affect the localization of the other. To determine if mutant LamC had an impact on the localization of the Drosophila B-type lamin, designated lamin Dm_0_ (lamDm_0_), immunohistochemistry using larval body wall muscles with antibodies to lamDm_0_ was performed. Larval body wall muscles expressing wild-type LamC showed the anticipated localization of lamDm_0_ at the nuclear periphery (Figure S3A). Larval muscles expressing LamC K521W showed lamDm_0_ staining at the nuclear periphery with enrichment in the nuclear lobulations (Figure S3A). Muscles expressing LamC R564P showed LamDm_0_ at the nuclear periphery. Thus, the results of LamC K521W suggest that mutations that affect A-type lamin localization can alter B-type localization.

A-type lamins interact with many nuclear envelope proteins, including those that make up nuclear pores [26,89,90]. To determine if LamC K521W and R564P altered the localization of nuclear pore proteins, larval body wall muscles expressing these mutants were stained with antibodies that recognize FG-repeat-containing nuclear pore proteins (NUPs). In muscles expressing wild-type LamC, the FG-repeat NUPs localized to the nuclear periphery as anticipated (Figure S3B). Similarly, muscles expressing LamC K521W showed FG-repeat NUPs confined to the nuclear periphery, even despite the nuclear envelope lobulations (Figure S3B). By contrast, muscles expressing LamC R564P showed FG-repeat NUPs at the nuclear envelope and in the cytoplasm (Figure S3B). Consistent with this finding, cytoplasmic NUPs have been observed in muscle tissue from individuals with LMNA-associated muscular dystrophy [91]. Thus, the mechanisms by which LamC R564P causes muscular dystrophy might be due to alterations in nuclear import/export as seen in mouse hearts under stress [92].

**Figure 3:**
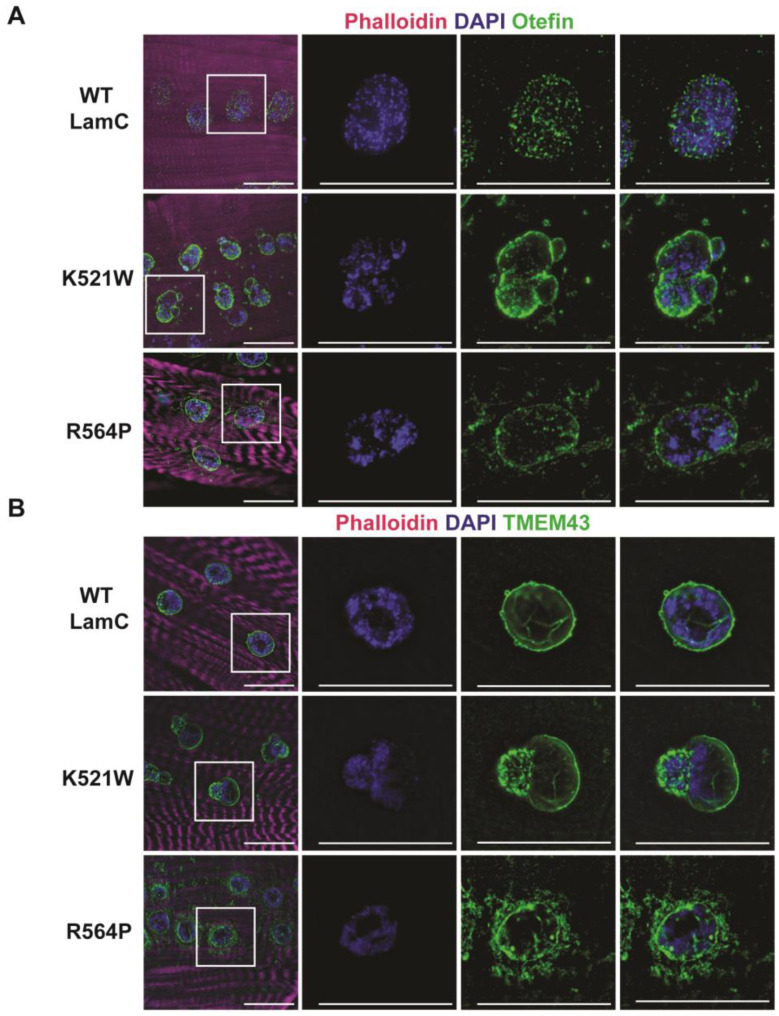
LamC K521W and R564P alter the localization of nuclear envelope proteins TMEM43 and Otefin. (**A**) Larval body wall muscles expressing either wild-type or mutant LamC were stained with phalloidin (magenta), DAPI (blue), and antibodies to Otefin (green). Note the cytoplasmic aggregation caused by both mutants. The white box outlines the magnified region shown on the right. (**B**) Larval body wall muscles expressing either wild-type or mutant LamC were stained with phalloidin (magenta), DAPI (blue), and antibodies to TMEM43 (green). Note the clustering of TMEM43 foci caused by LamC K521W and the cytoplasmic aggregation caused by LamC R564P. The scale bar represents 30 µm.

In mammalian cells, pathogenic A-type lamins disrupt the localization of the inner nuclear membrane protein emerin [93]. It is worthwhile to note that mutations in EMD, the gene encoding emerin, also cause EDMD [94]. To determine if LamC K521W and R564P alter the localization of a Drosophila emerin orthologue called Otefin [95], larval body wall muscles expressing either wild-type or mutant LamC were stained with antibodies against Otefin [55]. In muscles expressing wild-type LamC, Otefin was observed in fine grain puncta at the nuclear periphery, with a few speckles in the cytoplasm (Figure 3A). In muscles expressing LamC K521W and R564P, Otefin localized to the nuclear envelope and was also present in cytoplasmic foci (Figure 3A). 

Emerin and lamins interact with TMEM43, a multipass transmembrane protein that resides in the inner nuclear membrane [96,97]. Mutations in TMEM43 also cause EDMD-related myopathy [97,98]. To determine if LamC K521W and R564P altered the localization of the Drosophila orthologue of TMEM43 [99], larval body wall muscles were stained with a TMEM43 antibody (PA5-110497, Thermo Fisher Scientific). Muscles expressing wild-type LamC showed immunostaining confined to the nuclear periphery (Figure 3B). Muscles expressing LamC K521W showed TMEM43 staining at the nuclear periphery with enrichment in the nuclear lobulations (Figure 3B). By contrast, expression of LamC R564P showed TMEM43 at the nuclear periphery and aggregated in the cytoplasm (Figure 3B). Collectively, these results support prior findings that disruptions in the nuclear lamina alter inner membrane protein organization.

### 3.3. Mutant Lamins Have Different Effects on Larval Motility and Body Wall Size

**Figure 4:**
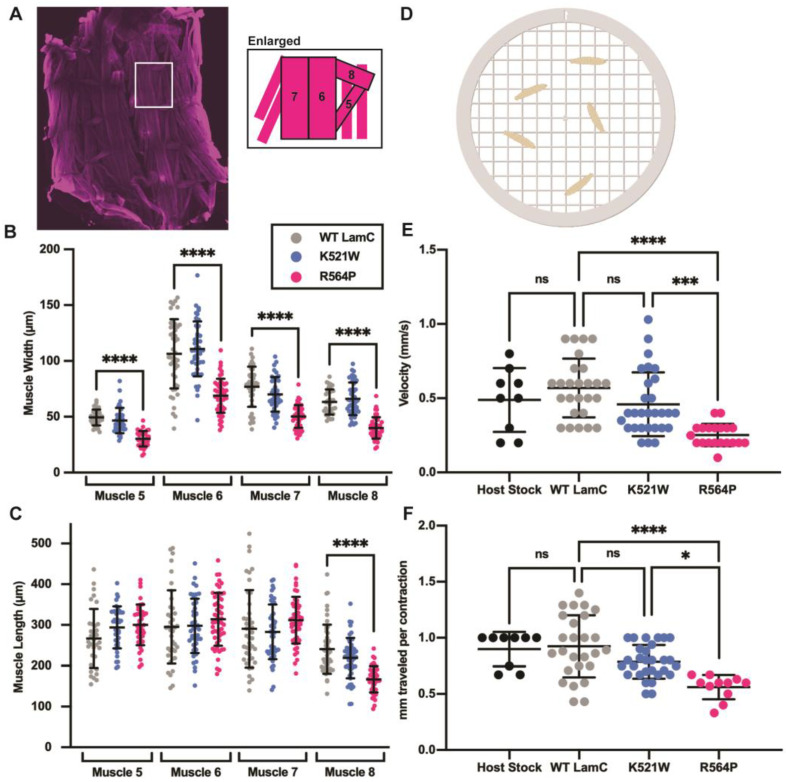
LamC R564P, but not LamC K521W, reduces larval motility and muscle size. (**A**) An image of a muscle filet from a hand-dissected third instar larvae stained with phalloidin (magenta) is shown. The diagram on the right shows the numbered muscles used for measurements. (**B**,**C**) The width and length of larval body wall muscles were measured for four different muscles diagrammed in A. Values are expressed as mean ± standard deviation with 33–53 muscles from 8–10 larvae analyzed per genotype. Statistical significance was determined using a one-way ANOVA analysis followed by Dunnett’s multiple comparisons test (GraphPad Prism version 9.5.0, GraphPad Software, San Diego, CA, USA) No statistical difference, ns; *p* > 0.05; * *p* < 0.05; ** *p* < 0.01; *** *p* < 0.001; **** *p* < 0.0001. (**D**) A diagram of the assay used to measure larval motility (see Materials and Methods for details) (created using BioRender.com; access date 11/2022). (**E**,**F**) Larval velocity and distance per larval contraction were measured by analyzing two-minute videos of larvae crawling on a thin layer of water in a petri dish. Values are expressed as the mean ± standard deviation, and 9–29 individual larvae were analyzed per genotype. Statistical significance was determined using a one-way ANOVA multiple comparisons analysis followed by Dunnett’s correction (GraphPad Prism version 9.5.0, GraphPad Software, San Diego, CA, USA). No statistical difference, ns; *p* > 0.05; * *p* < 0.05; *** *p* < 0.001; **** *p* < 0.0001.

During the dissection of body wall muscles for cytological analyses, it was apparent that muscles expressing LamC R564P were thin and extremely fragile compared to muscles expressing wild-type LamC. This prompted us to perform a quantitative morphometric analysis by measuring the same four larval body wall muscles (numbers 5, 6, 7, and 8, Figure 4A) from larvae of each genotype [74,100]. Larvae expressing wild-type LamC and LamC K521W had similar muscle widths and lengths (Figure 4B,C). Consistent with their apparently thin and fragile nature, muscles expressing LamC R564P had a significantly reduced width compared to muscles expressing wild-type LamC and LamC K521W (Figure 4B,C). By contrast, the muscle length was not dramatically altered, except for muscle eight. The reduction in muscle width might be due to a reduced expansion of the sarcomere during development as a result of a loss of protein homeostasis from altered nuclear import/export [73]. 

**Figure 5:**
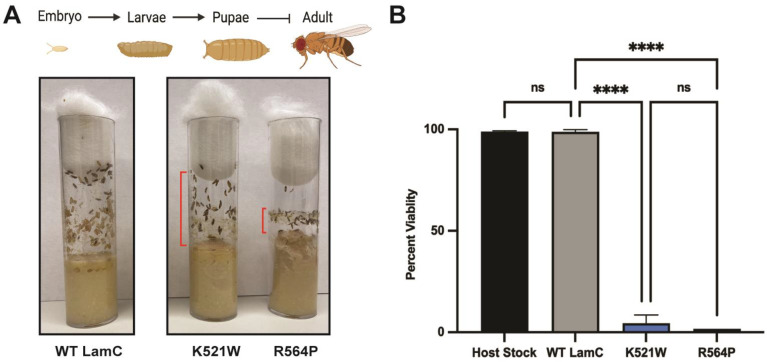
Larval body wall muscle-specific expression of mutant LamC reduces adult viability. (**A**) Graphical representation of the Drosophila lifecycle stages is shown at the top (created with BioRender.com, access date 12/2022). Vials containing larvae with larval body wall muscle-specific expression of either wild-type or mutant LamC are shown. Red bars indicate the larval movement up the side of the vial. (**B**) The percentage adult viability is represented as $\frac{\#\;living\;adults}{\#\;living\;adults+\#\;dead\;pupae}\times$ 100. The average percentage ± the standard deviation of the mean are shown. Total progeny (255–326) was counted from multiple individual crosses for each genotype. Statistical significance was determined using the Fisher’s exact test (GraphPad Prism version 9.5.0, GraphPad Software, San Diego, CA, USA). No statistical difference, ns; *p* < 0.001; **** *p* < 0.0001.

We wanted to determine if these cytological defects caused by mutant lamins altered muscle function. To achieve this, we performed larval motility assays by recording crawling behavior in a petri dish dampened with water (see Section 2) (Figure 4D). An analysis of the videos provided quantitative measurements of velocity (mm/s) and distance traveled per larval contraction (mm). Larvae expressing wild-type LamC showed no difference in velocity and distance per larval contraction compared to that of the host stock (Figure 4E,F). Larvae expressing LamC K521W also showed no difference in velocity and distance per larval contraction compared to larvae expressing wild-type LamC. By contrast, larvae expressing LamC R564P showed a significantly reduced larval velocity and distance per larval contraction when compared to those expressing wild-type LamC, consistent with the reduced size of the muscles. Moreover, observations of the culture vials supported the loss in motility for LamC R564P. During development, third instar larvae crawl up the side of the culture vial to pupate. Visual observation showed that larvae expressing LamC R564P did not crawl up the side of the vial to the same extent as larvae expressing wild-type LamC and LamC K521W (Figure 5A).

Since larval body wall muscles play a role in morphogenesis to adulthood [76,101,102], we assessed the impact of larval body wall muscle-specific expression of wild-type and mutant LamC on adult viability. Expression of wild-type LamC resulted in 98.8% of the progeny surviving to adulthood (Figure 5B). The development of larvae expressing LamC R564P resulted in only a 1.7% adult viability, consistent with the loss of muscle function (Figure 5B). Surprisingly, the development of larvae expressing LamC K521W resulted in only a 4.4% adult viability, despite the normal muscle morphology and larval motility. For both mutants, death occurred at the late pupal stage as observed by dark-color pupae that never eclosed as adults (Figure 5B). Therefore, despite the dramatically different effects on nuclear morphology and muscle function, both mutant lamins cause death at the pupal stage, perhaps via different molecular mechanisms.

### 3.4. Mutant Lamins Have Different Effects on Adipose Tissue

**Figure 6:**
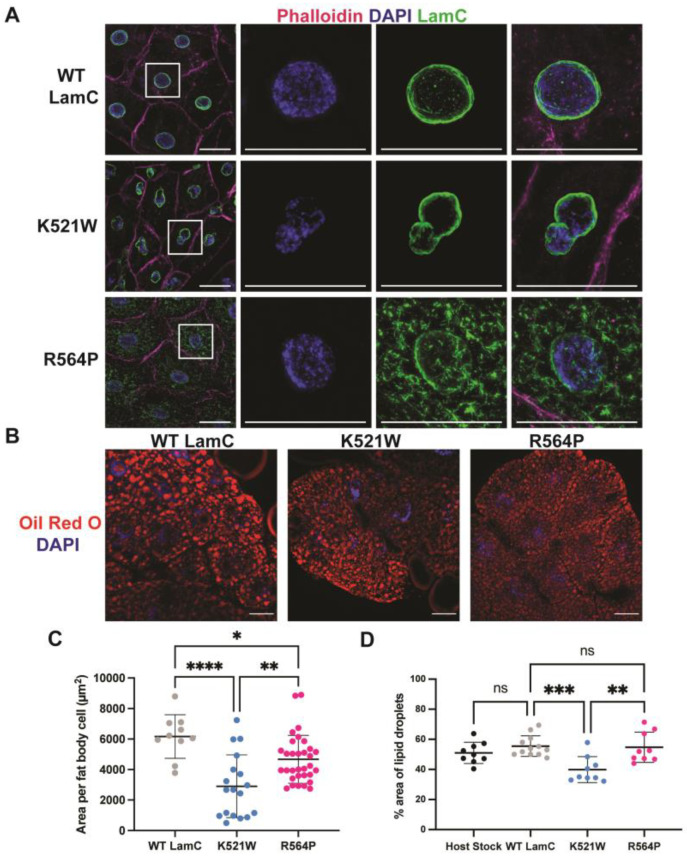
LamC K521W and R564P cause nuclear and cytoplasmic abnormalities in larval fat body tissue. (**A**) Larval fat body tissue expressing either wild-type or mutant LamC were stained with phalloidin (magenta), DAPI (blue), and antibodies to LamC (green). Note that LamC K521W localizes to the nuclear envelope and causes nuclear lobulations. By contrast, LamC R564P localizes within the cytoplasm and the nucleus remains spherical. (**B**) Larval fat body tissue expressing either wild-type or mutant LamC were stained with Oil Red O and DAPI. Scale bar represented 30 µm. (**C**) The average area per adipose cell for each genotype was determined and plotted. Note the reduced size of the fat body cells expressing LamC K521W compared to that of the control. (**D**) The percent area of lipid droplets was calculated and plotted. Three fat bodies from three larvae of similar age were analyzed per genotype. Error bars represent the standard deviation of the mean. Statistical significance was determined using ANOVA multiple comparisons analysis followed by Dunnett’s correction (GraphPad Prism version 9.5.0, GraphPad Software, San Diego, CA). No statistical difference, ns; *p* > 0.05; * *p* < 0.05; ** *p* < 0.01; *** *p* < 0.001; **** *p* < 0.0001.

Given that individuals with LMNA mutations encoding lamins A/C R482 and R527P exhibit FPLD2 or symptoms such as lipodystrophy [39,40,41], we wanted to examine the properties of these mutant lamins in Drosophila adipose tissue. To accomplish this, we utilized larval-fat-body-specific Gal4 drivers [P(Lsp2-Gal4.H3), stock #6357, and P[r4-Gal4}3, stock #33832, Bloomington Stock Center]. In larval fat body cells, wild-type LamC localized to the nuclear periphery as anticipated (Figure 6). Expression of LamC K521W caused an abnormal nuclear shape in fat cells that was reminiscent of that observed in larval body wall muscles (Figure 2 and Figure 6A). Despite this abnormal nuclear morphology, LamC retained its nuclear peripheral localization. By contrast, fat body cells expressing LamC R564P showed LamC immunostaining at the nuclear periphery and in foci throughout the cytoplasm (Figure 6). Thus, differences in LamC localization between the two lamins observed in larval body wall muscles were consistently seen in adipose tissue, suggesting common cellular mechanisms of disease. 

To determine if the mislocalization of mutant LamC altered properties of the adipose tissue, we examined the adipose cell size in larval fat bodies. The average area of the adipose cells was determined for each genotype. Adipose cells expressing LamC K521W were reduced in size compared to those expressing wild-type LamC and LamC R564P (Figure 6C). Given the reduced adipose cell size in larvae expressing LamC K521W, we wondered if there would be a reduced lipid content. Oil Red O is a fat-soluble dye used to stain neutral lipids and fat droplets. To examine the lipid content of the adipose tissue in larvae expressing either wild-type or mutant lamin, the fat bodies were dissected and stained with Oil Red O. A qualitative analysis of the resulting microscopic images indicated that the lipid droplets in adipose tissue expressing LamC K521W were smaller in size than those observed in adipose tissue expressing either wild-type LamC or LamC R564P (Figure 6B,D). The percentage area of fat-body-occupied lipid droplets was determined. The analysis revealed that 56% of the fat body tissue contained lipid droplets in larvae expressing wild-type LamC. By contrast, a 40% and 53% percentage area was lipid droplets in adipose tissue expressing LamC K521W and R564P, respectively. Only LamC K521W showed a statistically reduced area occupied by lipid droplets, suggesting an altered lipid metabolism.

### 3.5. Mutant Lamins Have Different Effects on Lifespan When Expressed in Cardiac Tissue

**Figure 7:**
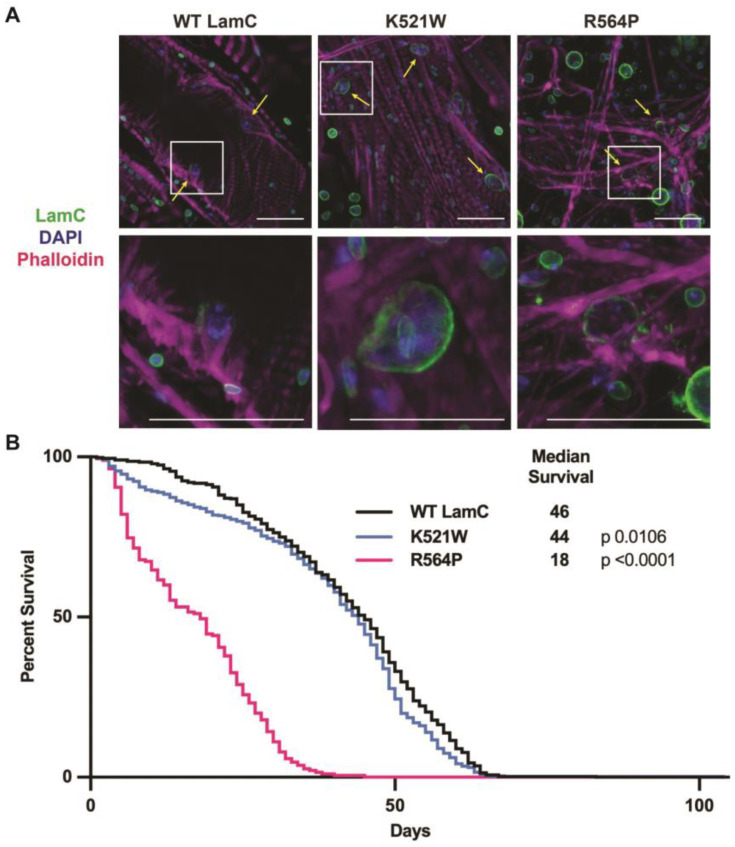
Cardiac-specific expression of LamC R564P, but not LamC K521W, reduces adult lifespan. (**A**) Drosophila hearts expressing either wild-type or mutant LamC were stained with phalloidin (magenta), DAPI (blue), and antibodies to LamC (green). The yellow arrows indicate cardiomyocytes. The scale bar represents 30 µm. (**B**) Adult longevity was determined for flies expressing either wild-type or mutant LamC in the heart. The graph shows the percent of living adults per day, which were maintained in fresh vials. The median survival time and the statistical analysis using the Mantel–Cox test was calculated using GraphPad Prism version 9.5.0, GraphPad Software, San Diego, CA, USA.

Individuals with *LMNA*-associated muscular dystrophy often develop dilated cardiomyopathy with conduction defects later in life [39,103]. To determine if the mutant lamins affect the *Drosophila* cardiac function, wild-type and mutant LamC were expressed in the dorsal vessel (heart tube) using the dHand4.2 Gal4 driver [62]. Immunohistochemistry was performed on three-day-old adults to examine LamC localization. Wild-type LamC and LamC K521W localized to the nuclear periphery (Figure 7A). By contrast, LamC R564P showed nuclear and cytoplasmic aggregation (Figure 7A), like that observed in larval body wall muscles (Figure 2A). Defects in the *Drosophila* heart function typically manifest as a shortened time to adult death [104,105,106]. The cardiac-specific expression of wild-type LamC and LamC K521W resulted in a median survival of 46 and 44 days, respectively (Figure 7B). By contrast, LamC R564P caused a severely reduced median survival of 18 days. Thus, LamC R564P, but not K521W, caused both skeletal muscle and cardiac defects that are accompanied by similar molecular defects.

## 4. Discussion

*Drosophila* is a powerful organism for modeling human disease, including neuromuscular disorders [107,108,109,110,111,112,113]. Such models have provided novel insights into disease mechanisms and identified potential drug targets and treatments [64,113,114,115,116,117,118]. In addition, *Drosophila* is one of a few selected model organisms used by the Undiagnosed Disease Network (UDN) to functionally test DNA sequence variants of uncertain significance in candidate disease genes (https://undiagnosed.hms.harvard.edu/). Here, *Drosophila* provided the ability to test mutant lamins using functional assays for larval body wall and cardiac muscles (Figure 4 and Figure 6).

According to the Universal Mutation Database (UMD; http://www.umd.be/; access date 12/2022) there are 185 reported cases of individuals with an *LMNA* mutation that results in p.R482W. The majority are cases of FPLD2 such as Priscilla; however, many of the individuals also have muscular dystrophy. The UMD shows 20 cases of individuals with an *LMNA* mutation that results in p. R527P with diagnoses of either EDMD2, EDMD2, or FPLD2, or limb-girdle muscular dystrophy type 1B (LGMD1B). Recall that Jill has EDMD2. This phenotypic heterogeneity is typical of laminopathies and strongly suggests the influence of genetic background. Our *Drosophila* models provided an opportunity to functionally test these two amino acid substitutions in a nearly identical genetic background, with the exception of the transgene insertion site. Our results indicate that R527P, but not R482W, inherently causes muscular dystrophy (Figure 4).

Based on the role of the larval body wall muscles in morphogenesis [64,81,82], it was not surprising that larvae expressing LamC R654P did not survive to adulthood. The muscles were thinner than wild-type and could not support larval motility (Figure 4). Surprisingly, LamC K521W also caused lethality at the pupal stage (Figure 5). This might be due to alterations in gene expression, as suggested by the changes in the abnormal organization of genomic DNA within the myonuclei (Figure 2A,C). In our prior studies, a loss of the N-terminal head domain of LamC (Figure 1A) caused changes in the expression of the steroid hormone ecdysone-regulated genes that govern morphogenesis [76]. It will be important to compare the transcriptomic profiles in muscles expressing each of the mutant lamins in the future.

Notable differences between the two mutant lamins were their intracellular localization and effects on nuclear morphology. LamC R564P localized to the cytoplasm and did not cause nuclear dysmorphology (Figure 2). By contrast, LamC K521W localized to the nucleus and caused nuclear lobulations. Nuclear blebbing and lobulations have been observed in different types of laminopathies [119,120,121]. Nuclear lobulations have been observed in *Drosophila* myonuclei expressing amino acid substitutions in the rod domain [31]. For LamC K521W and the rod domain substitutions, western analysis showed an additional higher molecular weight species recognized by the LamC antibody (Figure S1A) [31]. This increase in protein mobility is suggestive of post-translational modification (PTM). Lamins are known to be highly modified by PTMs, including phosphorylation upon nuclear envelope breakdown during mitosis [122,123]. In addition, lamins have a myriad of other modifications that include SUMOylation, glycosylation, farnesylation, methylation, and ubiquination [31,124,125,126,127]. In the future, it will be of interest to determine if such changes in molecular weight are observed in protein extracts from individuals possessing these amino acid substitutions. In laminopathy-cultured cells and mouse models, nuclear lobulations lead to transient nuclear envelope rupture and increased DNA damage [120,128]. Consistent with this finding, increased DNA damage is observed in human muscle tissue from individuals with *LMNA-*associated muscular dystrophy [120].

In addition to providing structural support for the nucleus, the lamin meshwork acts as a scaffold that helps to organize many proteins that are associated with the nuclear envelope, including nuclear pores [21,129,130] (Figure 3 and Figure S3). The muscle-specific expression of LamC R564P causes the cytoplasmic localization of nuclear pore proteins (Figure S3). Consistent with this finding in *Drosophila*, cytoplasmic NUPs are also observed in human muscle biopsy tissue from individuals with *LMNA*-associated muscular dystrophy [91], demonstrating clinical relevance. Given that some mutant lamins cause cytoplasmic nuclear pore distribution, whereas others do not, there might be different mechanisms of pathogenesis among individuals with skeletal muscle laminopathies [31,91,116].

**Figure 8:**
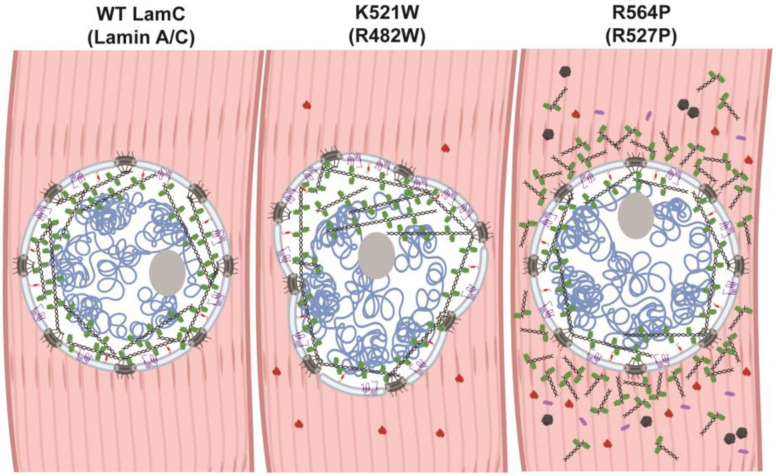
A model showing the differential effects of the two mutant lamins on the localization of nuclear proteins. (**Left**) Muscle expressing wild-type LamC (green) exhibit a lamin meshwork that lines the inner nuclear membrane. Nuclear pores (dark gray), TMEM43 (purple) and Otefin (red) are confined to the nuclear envelope. The DNA is represented in blue and the nucleolus organizer in gray. (**Middle**) Muscle expressing LamC K521W exhibits an uneven distribution of the lamin meshwork and nuclear lobulations with nuclear pores and TMEM43 confined to the nuclear envelope. By contrast, a portion of Otefin is mislocalized to the cytoplasm. As a result of the nuclear shape change, the genomic DNA (blue) has an atypical distribution. (**Right**) Muscles expressing LamC R564P exhibit abnormal localization of LamC, FG-repeat nuclear pore proteins, TMEM43, and Otefin throughout the cytoplasm.

The mechanisms by which *LMNA* mutations cause muscular dystrophy and lipodystrophy are not well understood. It is possible that different mutations cause disease by different mechanisms. Public databases that list pathogenicity predictions based on evolutionary sequence conservation and biochemical properties of amino acids predict that both substitutions are pathogenic [ClinVar, Leiden Open Variant Database (LOVD^3^), Genome Aggregation Database (gnomAD); access date 12/2022] [131,132,133]. In addition, ddGUN [134] can be used to calculate the thermodynamics of protein unfolding and predict that both amino acid substitutions in lamin A/C destabilize the Ig-like fold domain in comparison to that of the wild-type. Destabilization of the Ig-like fold could cause lamin malfunction via many mechanisms. The Ig-like fold domain interacts with a plethora of partner proteins and plays a role in lamina meshwork assembly [135]. Thus, destabilization of the Ig-like fold might lead to ineffective interactions that cause the formation of a defective meshwork, contributing to the nuclear shape changes as observed for LamC K521W (Figure 8). Destabilization of the Ig-like fold domain could also alter the availability of the nuclear localization sequence (Figure 1A) to the nuclear import machinery and block posttranslational modifications required for proteostasis [136], which could explain the cytoplasmic accumulation observed for LamC R564P in both muscle and adipose tissue. A cytoplasmic accumulation of proteins is often a sign of inefficient autophagy. Consistent with this observation, genetic manipulations and pharmacological treatments that increase autophagy suppress muscle defects in *Drosophila* and mouse models of muscle laminopathies [137,138].

The relationship between autophagy and lipid metabolism is complex [139,140]. In *Drosophila* larval fat body tissue, we observed a reduction in the percentage area of lipid droplets in larvae expressing LamC K521W compared with larvae expressing LamC R564P and wild-type LamC (Figure 6B,D). In our studies, the mutant LamC was exclusively expressed in the fat body cells, suggesting that the reduced area of lipid droplets caused by LamC K521W was cell-intrinsic and not due to effects from other tissues as they were wild-type. This reduction in lipid content might indicate alterations in lipid production and/or degradation. Consistent with the idea of an increased degradation, cultured cells possessing *LMNA* R482W show a reduced fat production and increased lipolysis [139].

In summary, we developed *Drosophila* models that show dominant effects of mutant lamins like that observed in humans. Furthermore, these defects recapitulate the skeletal muscle physiology of two individuals, Jill and Priscilla, studied here. These models show that the mutant lamins possess different patterns of subcellular localization in muscle and have opposing effects on nuclear morphology and muscle function (Figure 2, Figure 3, Figure 4, Figure 7 and Figure 8). Furthermore, defective phenotypes observed in the larval body wall muscles were also observed in cardiac tissue (Figure 7), suggesting common pathological mechanisms in both muscle types. In the future, these models will be useful for testing genetic and pharmacological interventions to mitigate the muscle and adipose tissue defects, providing opportunities for treatments.

## Data Availability

Not applicable.

## Supplementary Materials

The following supporting information can be downloaded at: https://www.mdpi.com/article/10.3390/cells12081142/s1. Figure S1. Western analysis was used to determine the levels of LamC in transgenic and control lines. (**A**) Proteins extracted from larval body wall muscles expressing wild-type or mutant LamC were separated by size on an SDS/PAGE gel, transferred to membrane, which was then stained with antibodies to LamC. Green arrows indicate LamC products at the anticipated molecular weight (~70 kD). The green arrow with the asterisk indicates a higher molecular weight species that might result from post-translational modification. The modification on this isoform is unknown. However, it is worthwhile to note that lamins are heavily post-translationally modified [124,125,126,127] and a similar higher molecular weight band has been observed in our prior studies for substitutions in the rod domain [31]. The black asterisk indicates faint bands present among all genotypes. The LamC K521Q (5m-1) sample was not used in the studies reported here. (**B**) The membrane shown in panel A was incubated with an antibody to GAPDH as a control for protein loading. The red arrow indicates a band at the anticipated molecular weight of GAPDH. The black asterisk indicates bands recognized in all genotypes. (**C**) Relative amounts of LamC protein in the transgenic lines and control host stock are shown. LamC protein levels were normalized to GAPDH levels and made relative that of LamC in the wild-type sample. The results from three independent biological samples were averaged and plotted. Statistical significance was determined using a one-way ANOVA analysis followed by Dunnett’s multiple Comparisons test (GraphPad Prism version 9.5.0, GraphPad Software, San Diego, CA). ns, nonsignificant; ** p >* 0.05; **** p <* 0.001. Figure S2. Endogenous Lamin C localizes to the nuclear periphery in the absence of Gal4 expression. Larval body wall muscles possessing either wild-type LamC, LamC K521W, or R564P were stained with phalloidin (magenta), DAPI (blue), and antibodies to LamC (green). Without GAL4 expression, these transgenes should not be expressed. Note that the anti-LamC antibody detected LamC endogenous LamC localization at the nuclear periphery in all three genotypes. The scale bar represents 30 µm. Figure S3. Mutant LamC alters the localization of lamDm_0_ and FG-repeat containing nuclear pore proteins (NUPs). (**A**) Larval body wall muscles expressing either wild-type or mutant LamC were stained with phalloidin (magenta), DAPI (blue), and antibodies to lamDm_0_ (green). The scale bar represents 30 µm. Note that LamC K521W alters the nuclear envelope distribution of lamDm_0_. (**B**) Larval body wall muscles expressing either wild-type or mutant LamC were stained with phalloidin (magenta), DAPI (blue), and antibodies to FG-repeat containing NUPs. Note that the NUPs retained nuclear peripheral localization in muscle expressing LamC K521W; however, the FG-repeat containing NUPs localized to both the nuclear periphery and the cytoplasm in muscles expressing LamC R564P. The scale bar represents 30 µm.
