# Supplementary material for: Drosophila Models Reveal Properties of Mutant Lamins That Give Rise to Distinct Diseases
Source: Cells. 2023 Apr 12;12(8):1142. doi: 10.3390/cells12081142 (PMC10136830; doi:10.3390/cells12081142)
Supplement: Supplementary file 1 [file cells-12-01142-s001.zip › cells-2212235-supplementary.pdf]

Supplementary Materials

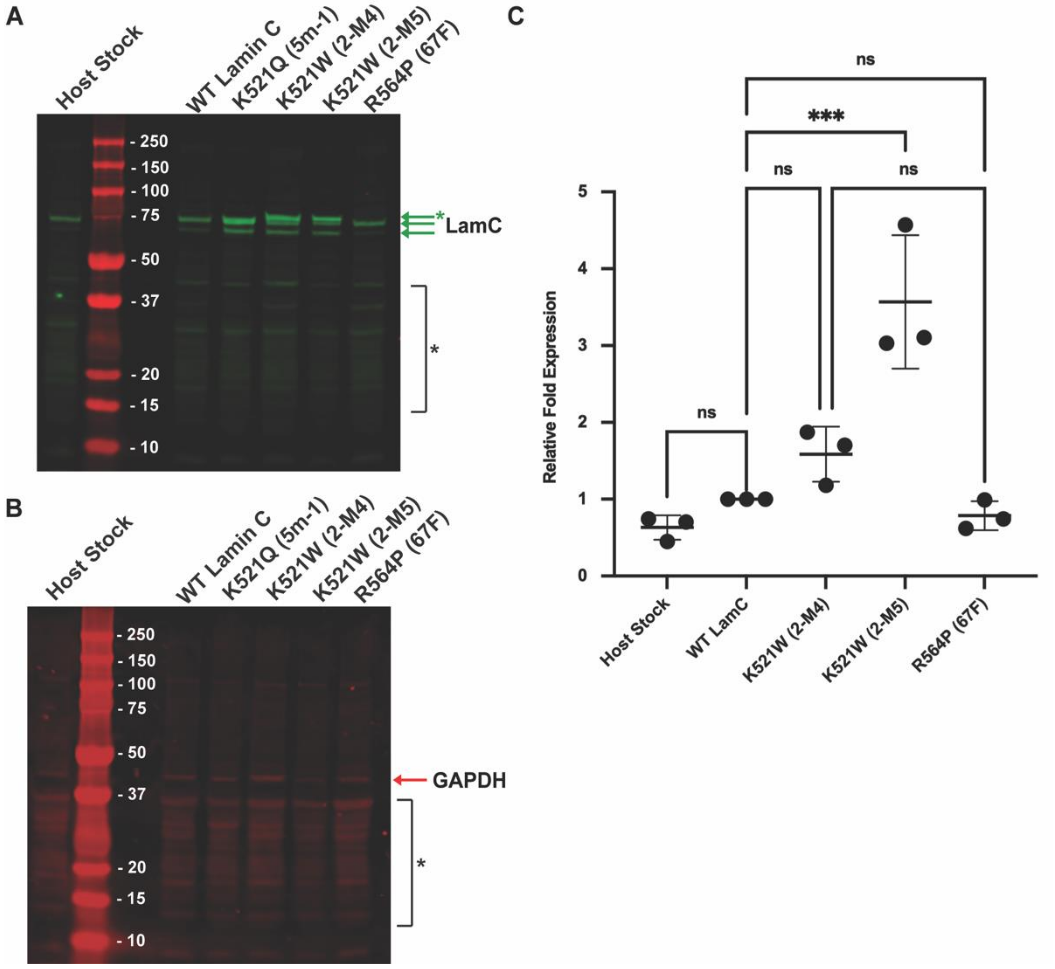

Figure S1. Western analysis was used to determine the levels of LamC in transgenic and control lines. (**A**) Proteins extracted from larval body wall muscles expressing wild-type or mutant LamC were separated by size on an SDS/PAGE gel, transferred to membrane, which was then stained with antibodies to LamC. Green arrows indicate LamC products at the anticipated molecular weight (~70 kD). The green arrow with the asterisk indicates a higher molecular weight species that might result from post-translational modification. The modification on this isoform is unknown. However, it is worthwhile to note that lamins are heavily post-translationally modified [124–127] and a similar higher molecular weight band has been observed in our prior studies for substitutions in the rod domain [31]. The black asterisk indicates faint bands present among all genotypes. The LamC K521Q (5m-1) sample was not used in the studies reported here. (**B**) The membrane shown in panel A was incubated with an antibody to GAPDH as a control for protein loading. The red arrow indicates a band at the anticipated molecular weight of GAPDH. The black asterisk indicates bands recognized in all genotypes. (**C**) Relative amounts of LamC protein in the transgenic lines and control host stock are shown. LamC protein levels were normalized to GAPDH levels and made relative that of LamC in the wild-type sample. The results from three independent biological samples were averaged and plotted. Statistical significance was determined using a one-way ANOVA analysis followed by Dunnett's multiple Comparisons test (GraphPad Prism version 9.5.0, GraphPad Software, San Diego, CA). ns, nonsignificant; * $p > 0.05$; *** $p < 0.001$.

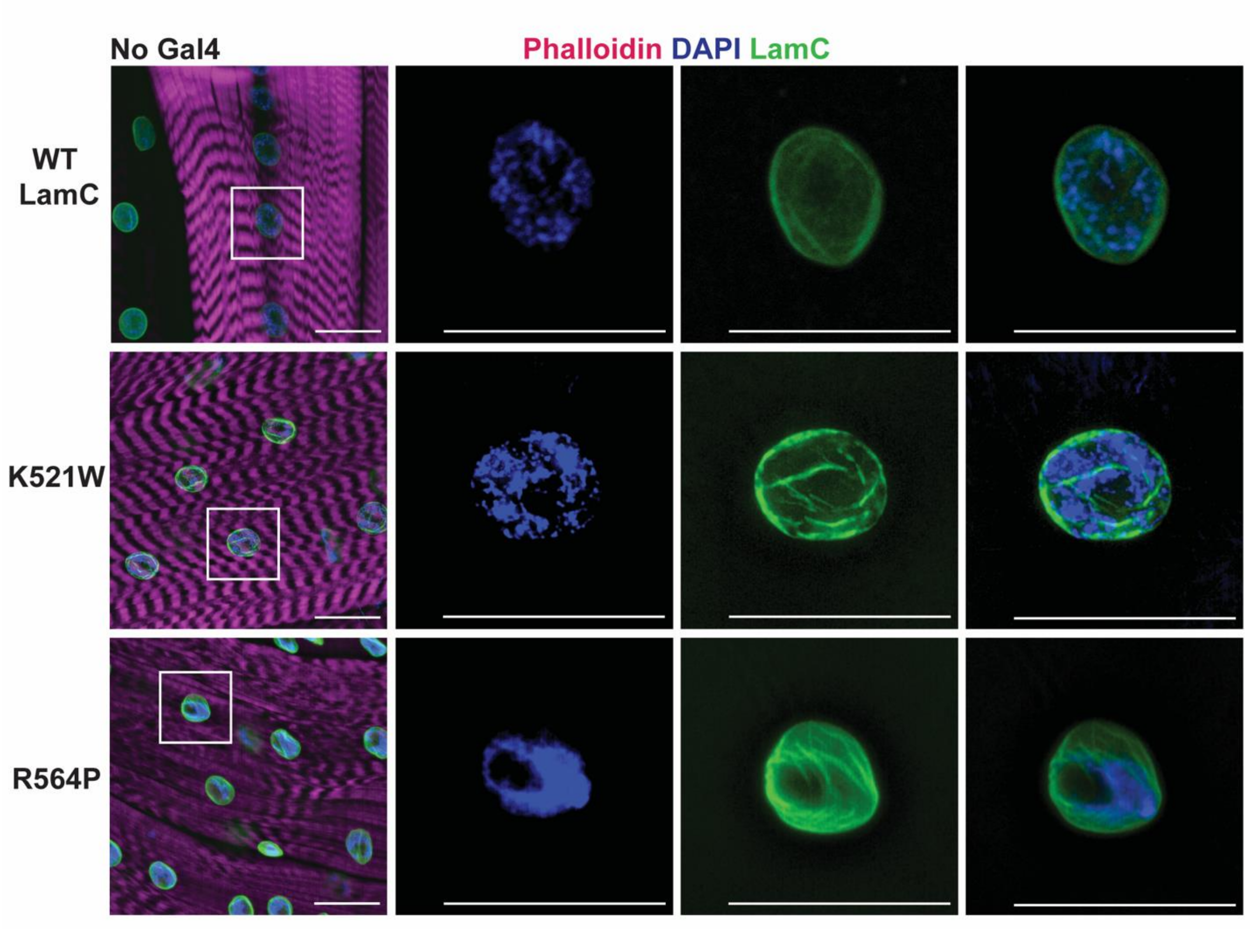

Figure S2. Endogenous Lamin C localizes to the nuclear periphery in the absence of Gal4 expression. Larval body wall muscles possessing either wild-type LamC, LamC K521W, or R564P were stained with phalloidin (magenta), DAPI (blue), and antibodies to LamC (green). Without GAL4 expression, these transgenes should not be expressed. Note that the anti-LamC antibody detected LamC endogenous LamC localization at the nuclear periphery in all three genotypes. The scale bar represents 30 μm.

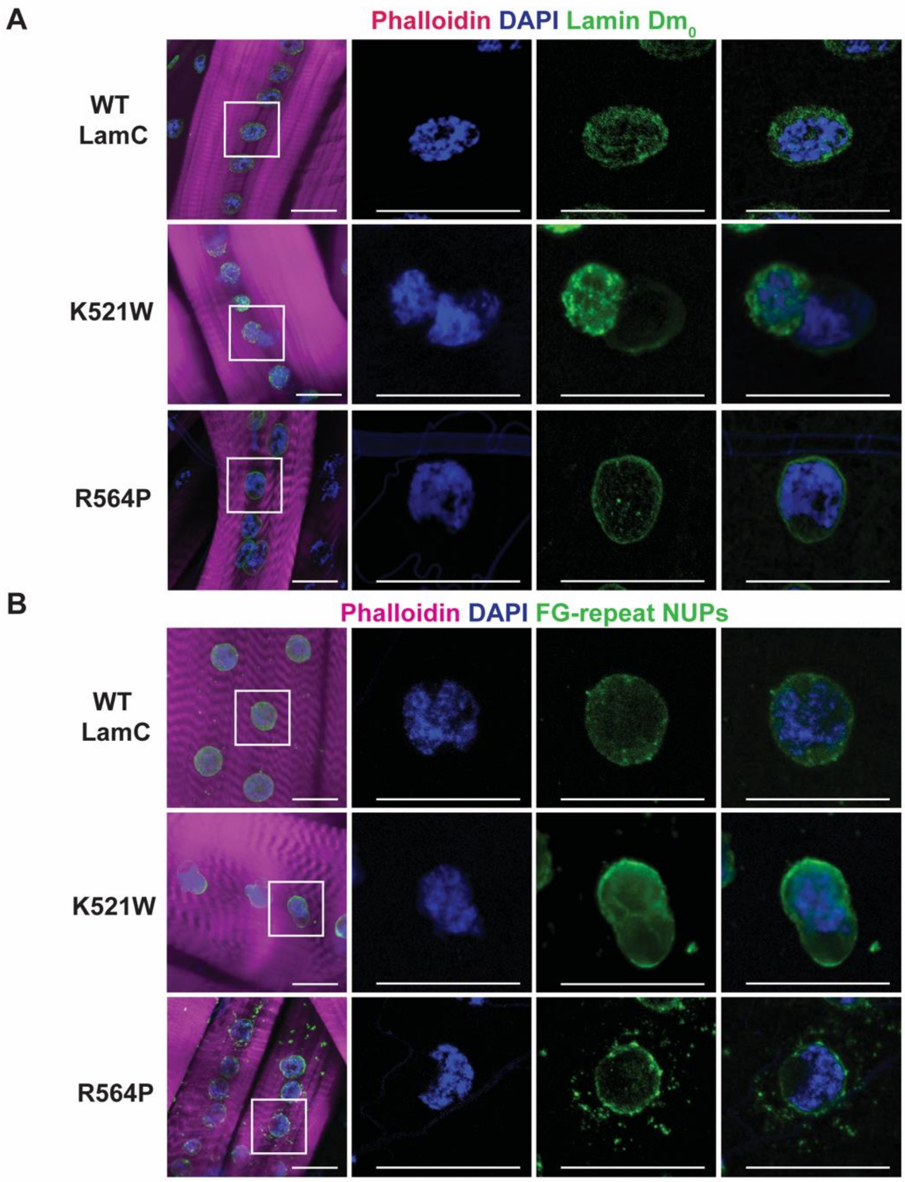

Figure S3. Mutant LamC alters the localization of $lamDm_0$ and FG-repeat containing nuclear pore proteins (NUPs). (**A**) Larval body wall muscles expressing either wild-type or mutant LamC were stained with phalloidin (magenta), DAPI (blue), and antibodies to $lamDm_0$ (green). The scale bar represents 30 μm. Note that LamC K521W alters the nuclear envelope distribution of $lamDm_0$. (**B**) Larval body wall muscles expressing either wild-type or mutant LamC were stained with phalloidin (magenta), DAPI (blue), and antibodies to FG-repeat containing NUPs. Note that the NUPs retained nuclear peripheral localization in muscle expressing LamC K521W; however, the FG-repeat containing NUPs localized to both the nuclear periphery and the cytoplasm in muscles expressing LamC R564P. The scale bar represents 30 μm.
